# Tocolytic Treatment for the Prevention of Preterm Birth from a Taiwanese Perspective: A Survey of Taiwanese Obstetric Specialists

**DOI:** 10.3390/ijerph19074222

**Published:** 2022-04-01

**Authors:** Howard Hao Lee, Chang-Ching Yeh, Szu-Ting Yang, Chia-Hao Liu, Yi-Jen Chen, Peng-Hui Wang

**Affiliations:** 1 Department of Obstetrics and Gynecology, Taipei Veterans General Hospital, Taipei 112, Taiwan; l.harvee@gmail.com (H.H.L.); ccyeh@vghtpe.gov.tw (C.-C.Y.); styang6@vghtpe.gov.tw (S.-T.Y.); mikeliuu@gmail.com (C.-H.L.); chenyj@vghtpe.gov.tw (Y.-J.C.); 2 Department of Obstetrics and Gynecology, National Yang Ming Chiao Tung University, Taipei 112, Taiwan; 3 Institute of Clinical Medicine, National Yang Ming Chiao Tung University, Taipei 112, Taiwan; 4 Department of Medical Research, China Medical University Hospital, Taichung 404, Taiwan; 5 Female Cancer Foundation, Taipei 112, Taiwan

**Keywords:** preterm birth, preterm labor, tocolysis, tocolytics

## Abstract

Preterm birth represents a great burden to the healthcare system, resulting in the consideration for the use of tocolytic therapy to provide a “better time” for delivery in order to buy time to accelerate fetal lung maturity, thereby minimizing prematurity-related morbidity and mortality. However, the benefits and potential side effects and risks of tocolytic treatment for preterm birth should be carefully balanced. Although many countries and societies provide guidelines or consensuses for the management for preterm birth, there is no standardized national guideline or consensus in Taiwan. As such, great heterogeneity is suspected in preterm labor management, contributing to the uncertainty of attitudes and practice patterns of obstetric specialists in Taiwan. This study attempts to understand the attitudes and practice patterns regarding tocolytic therapy in Taiwan. A paper-based survey was conducted at the 2020 Taiwan Society of Perinatology Conference on 8 December 2020, exploring how obstetric specialists would use tocolytics under nine different clinical scenarios, such as a short cervix, preterm labor, maintenance tocolysis, preterm premature rupture of membranes, etc. Three hundred ten specialists attended the conference, and 77 responded to the survey with a response rate of 24.8%. According to the survey, many of these specialists would prescribe tocolytics for less evidence-based indications, including 22% for abdominal tightness, 46% for a short cervix, 60% for maintenance tocolysis, and 89% for repeat tocolysis, with the preferred first line medication being ritodrine and nifedipine. We concluded that tocolysis is widely accepted and practiced in Taiwan. More research is needed to include Taiwan-specific economic and cultural factors as well as associated adverse effects and patients’ outcomes.

## 1. Introduction

Preterm birth is still a major challenge for obstetricians worldwide and is associated with neonatal complications, such as respiratory distress syndrome (RDS), intraventricular hemorrhage (IVH), necrotizing enterocolitis (NE), and retinopathy of prematurity [1,2,3,4,5]. According to the Global Health Observatory by the World Health Organization, complications of prematurity are a leading cause of death under 5 years of age, with 964,366 deaths in 2017 alone, accounting for 17.8% of the under-5-mortality globally [6,7]. Furthermore, preterm birth is a great burden to the healthcare system as well as a source of substantial financial and psychological hardship [8,9,10].

According to the World Health Organization (WHO) statistics in 2010, the preterm birth rate in Taiwan stood at 9% [11]. More recent data from Taiwan’s Birth Reporting System collated by the Health Promotion Administration (HPA) shows a slow but steady upward trend in preterm births in the past 10 years, with the latest preterm birth rate rising to 10.4% in 2019 [12]. However, it is not known whether the rising rate is due to spontaneous preterm births or to medically indicated preterm delivery. A study looking at preterm birth rates in Taiwan from 2004 to 2013 showed that even though medically indicated preterm birth rates surpassed spontaneous preterm birth rates in 2005, there has been a general upward trend in both [13].

Currently, preterm labor is treated by different types of pharmacological agents. However, the usage of tocolytic agents for preterm labor is disputed among obstetricians worldwide, and professional organizations have established various guidelines which vary greatly in their recommendations [14]. To emphasize, preterm birth is still a challenge to researchers studying its basic mechanisms, and though there have been some recent headways into its molecular pathways and prediction models [15], the question of when to start tocolytic therapy and for how long remains a common but difficult decision for clinicians. Currently, it is generally recommended that tocolysis be limited to the occurrence of preterm labor between the gestational ages (GA) of 24 weeks and 30 weeks or to those over 30 weeks of GA accompanied with cervical length <15 mm or 20 mm [4,16,17,18,19,20,21,22,23,24]. The most acceptable strategy is acute tocolysis or 48 h of tocolytic agent use for steroid and magnesium sulphate (MgSO4) administration, as well as to gain time for maternal–fetal transfer [25,26,27,28]. As of now, it is still uncertain whether the maintenance and repeating of tocolysis will improve neonatal outcomes [28,29,30,31,32,33,34]. Indeed, a systematic review analyzing 16 different international guidelines, including those from the WHO, the International Society of Ultrasound in Obstetrics and Gynecology (ISUOG), the United States of America (USA), Canada, the United Kingdom (UK), Belgium, France, Japan, China, Australia, and New Zealand, etc., showed that most guidelines agreed on acute tocolysis for threatened preterm labor and recommended against long-term tocolysis [20].

Tocolytics themselves are also riddled with side effects and present with many controversies. Terbutaline, a beta-mimetic, was given a black-box warning by the USA Food and Drug Administration (FDA) due to its side effects, such as cardiac arrhythmias and pulmonary edema [35]. Ritodrine, another betamimetic, has also been removed from the American market [36,37]. In Taiwan, the Taiwan FDA (TFDA) had considered prohibiting the tocolytic ritodrine in 2020 based on its adverse events, even though it was approved for tocolysis only recently in 2013 [38]. However, the aforementioned recommendation or policy of the TFDA did not convince the majority of the obstetricians in Taiwan. This resulted in a statement issued by the Taiwanese Association of Obstetrics and Gynecology (TAOG) that did not outright ban the medication but did emphasize the “the need for stricter screening for inherent medical illnesses”, such as underlying arrhythmia and abnormal thyroid function, before prescribing, as well as for closer surveillance for adverse effects during its use [39]. The TFDA and the Taiwan Drug Relief Foundation also issued a warning for health personnel regarding another tocolytic medication, indomethacin. Notably, it cautioned against prescribing indomethacin for GA over 30 weeks, which is stricter than the generally accepted GA 32 weeks due to premature closure of the patent ductus arteriosus [40].

Currently, there are no studies looking at the tocolysis practice patterns in Taiwan. A great heterogeneity is suspected, as there is no standardized national guideline regarding preterm labor management. This study attempts to understand the attitudes and practice patterns of tocolytic therapy in Taiwan.

## 2. Materials and Methods

We conducted a paper-based survey at the 2020 Taiwan Society of Perinatology Conference on 8 December 2020. All attendees to the conference were given a survey and included in the study should they have chosen to participate. Since training physicians, such as obstetric fellows and residents, could also attend, they were also given a survey to fill out if they were so inclined but were not ultimately included in the final analysis of this study. Only certified obstetric specialists were included. Ethics approval was given by the Taipei Veterans General Hospital Institutional Review Board (IRB), IRB number 2020-12-002BC, on 7 December 2020.

The survey was composed of multiple-choice questions and divided into two sections. The first section was questions regarding basic demographics, and the second consisted of questions detailing tocolytic use in nine different clinical scenarios. All of the scenarios pertained to a pregnant woman, gravida 1 and parous 0 (G1P0), with no medical or surgical history, currently at GA 26 + 0 weeks. She did not smoke, chew betel nuts, or drink, and all prenatal check-ups were up to date, with no chromosomal abnormalities or congenital anomalies noted. The clinical scenarios were as follows: 1. Abdominal tightness, 2. Short cervix, 3. Preterm contractions, 4. Preterm labor, 5. Maintenance tocolysis, 6. Repeat tocolysis, 7. Preterm premature rupture of membrane (PPROM), 8. PPROM with contractions, and 9. PPROM with preterm labor. For each of the scenarios, respondents were asked whether they would prescribe tocolytic therapy, would not recommend but would still prescribe tocolytic therapy if the patient has a strong desire for tocolytics, or would not recommend and would refuse to prescribe tocolytics. If tocolytics were prescribed, follow-up questions regarding the choice of medication were then asked (Table 1).

A Fischer exact test and Chi-squared test were used for categorical variables using SPSS 19.0.0 for Windows, copyright 1989, 2010, Chicago, IL, USA.

## 3. Results

Of the 310 specialists who attended the conference, 77 surveys were recovered to a response rate of 24.8%. The demographics of the respondents are shown in Table 2. Just over half of respondents practiced at medical centers (57.1%), while the rest came from regional hospitals (19.5%), municipal hospitals (10.4%), and local clinics (13.0%).

Respondent’s recommendations for tocolysis are shown in Table 3. A majority of specialists would recommend and prescribe tocolytics for a short cervix (60%), preterm contractions (88%), preterm labor (95%), maintenance tocolysis (60%), repeating tocolysis (89%), PPROM (65%), PPROM with contractions (83%), and PPROM with preterm labor (82%). As for abdominal tightness without any other symptoms, most would not recommend tocolysis, but 42% would still prescribe tocolytics if the patient strongly desired. The first-line tocolytic preferred for almost all of the scenarios was ritodrine, either mediated by oral or intravenous (IV) forms, and oral nifedipine was a second choice apart from in preterm contractions, where 44% of respondents chose nifedipine as the first choice.

Two demographic variables were collected, namely affiliated practice type and years practicing as specialists, as shown in Table 4. There was no significant difference with regards to recommendations for tocolysis between different affiliated practice types. No significant difference was found between the years practicing as obstetric specialists for most of the scenarios apart from PPROM with contractions (*p* = 0.005) and PPROM with preterm labor (*p* = 0.021). In both instances, obstetric specialists with 16–20 years and 21–25 years of experience did not recommend tocolysis and would not prescribe it, even if the patient requested it at a higher rate than other groups (*p* = 0.005 and 0.021, respectively). The same trend could be seen for PPROM without contractions or cervical effacement, although it did not reach statistical difference.

A further analysis using medical center and non-center affiliations did not show any significant differences, as shown in Table 5. If using 15 years as a cut-off, obstetric specialists with >15 years of experience would not prescribe tocolysis at a higher rate than those with less experience (*p* = 0.034). The same was true for PPROM with contractions (*p* = 0.030).

## 4. Discussion

Tocolysis is widely practiced and accepted in Taiwan, and indications include well-supported settings, such as preterm labor and PPROM. However, we found that tocolytics are also commonly recommended in settings less supported by international guidelines or less backed by scientific evidence, as in the case of a short cervix (61%), maintenance tocolysis (60%), and repeat tocolysis (89%). There was no significant difference in the practice patterns between the different levels of affiliated practice. Obstetric specialists practicing for 16–25 years recommended no tocolysis when it came to PPROM with contractions and PPROM with preterm labor at a higher rate than other groups (*p* = 0.005 and 0.021 respectively). However, due to the smaller sample size and no clear trend upon further analysis using 15 years as a cut off, the significance of this finding remains to be debated. In conclusion, the above findings would suggest that tocolysis in settings less well-supported by scientific evidence is accepted, in general, as part of obstetric practice in Taiwan.

There have been many similar studies conducted in countries around the world [22], including in Australia [41], Austria [30], Canada [42], France [18,43], New Zealand [41], UK [44], Germany [4], and USA [45]. All of these studies found that tocolysis practice patterns varied widely between practitioners and that not all obstetricians prescribed according to scientific evidence, as is the case in Taiwan, and this study attempted to address this concern. Furthermore, establishing a standardized national guideline does not always translate into clinical practice. A study in the UK comparing preterm labor management before and after the National Institute of Health and Excellence (NICE) published a guideline for Preterm labour and birth (NICE guideline 25) found that although there appears to be increased consensus in cervical length screening, there remain great variations in other areas of preterm management, such as tocolytic use [46]. More notably, a 2020 study in France looked at practice patterns before and after national guidelines regarding tocolysis were published in 2018 and found that guideline adherence was generally low, with little improvement after dissemination of the guideline [18].

Several factors can be hypothesized to contribute to this finding in Taiwan. Low fertility rates and an aging population coupled with government support for encouraging childbearing may play a role in more aggressive measures to treat and prevent preterm birth [47]. Cultural factors and representation in the media may also be explored. Lastly, a fear of malpractice lawsuits and patient dissatisfaction may also be considered. Indeed, patient input plays a large part in an obstetrician’s decision making, as was seen in our study. When considering tocolytics for simply abdominal tightness, 42% of respondents would not recommend tocolytics but would still prescribe it if the patient strongly desired. Similarly, 26% of obstetricians would prescribe it upon patient request when it came to maintenance tocolysis. This was also found in a similar American study by Fox et al. [45].

As for the choice of tocolytic, ritodrine, whether in oral or intravenous form, was the preferred medication, apart from in preterm contractions, where nifedipine (44%) was the first choice. However, if taking into account both oral and intravenous forms of ritodrine, it would amount to 53.4%, still surpassing all other tocolytics. This is despite the TAOG recommendations for a more complete survey for arrhythmias and thyroid dysfunction, as well as to use an IV pump for more accurate dosing, both of which result in higher barriers to prescription and, in theory, preclude out-patient use [39].

More notably, 48.3% of respondents would use ritodrine for maintenance tocolytic therapy. This is in contrast to the decreasing support for ritodrine use in the USA and Europe, where ritodrine is no longer recommended as the first-line tocolytic, and more in line with obstetric practice in Japan [48]. A retrospective cohort study in 2019 found that of the 373,858 Japanese women identified with threatened preterm labor, 36% were treated with ritodrine, and in 82.8% of the patients, ritodrine was continued for >48 h. In the same study, long-term tocolysis was associated with an increased incidence of maternal adverse effects with no mention of improved perinatal outcomes, showing the need for further research [49].

As of now, this is the only study to have explored the attitudes and practice patterns regarding tocolytic therapy in Taiwan. Even though this study did not directly analyze clinical practice, we feel that this study accurately reflects obstetric practice in Taiwan. Compared to the use of national databases where there is no strict definition of threatened preterm labor, whether it be simply abdominal tightness or regular contractions with cervical dilation, the way the survey was designed was able to draw out the nuances of obstetrical practice in a much more structured way. Furthermore, our decision to exclude non-obstetric specialists from the analysis was to further ensure that current practice patterns were reflected in this study.

In terms of respondent demographics, over half (57%) of the respondents were specialists from medical centers. This is in direct contrast to the true demographics of obstetric specialists in the whole of Taiwan, where, according to the Taiwan Medical Association 2020 statistics, only 25% of obstetric-gynecologists practice in medical centers and where over half practice in local clinics [50]. However, the Taiwan Medical Association does not differentiate between gynecologists and obstetricians. Many obstetrics and gynecology specialists in local clinics practice gynecology exclusively. This can be seen in the 2019 Statistics of the Birth Reporting System, where 72% of births took place in hospitals, while only 28% took place in local clinics [12]. We feel that the place of birth better reflects the affiliated practice types of obstetricians in Taiwan and is comparable to the demographics of the respondents in this study.

Limitations to be acknowledged include the possibility of selection bias, as not all obstetricians in Taiwan are members of the Taiwanese Society of Perinatology, and not all members attended the conference. Furthermore, not all attendees chose to participate in the survey. Therefore, the obstetricians sampled in this study may not have been completely reflective of all obstetricians in Taiwan. Furthermore, atosiban is out-of-pocket in Taiwan and not covered by the Taiwan National Health Insurance (NHI), which is the main source of medical care for the general population in Taiwan [51,52,53,54,55,56,57,58,59,60], and it may have influenced the decision making in clinical practice. The length of the tocolytic used in PPROM was also not included in the survey, so questions regarding the balance of tocolysis and intrauterine infection risk could not be answered. Similarly, specific reasons for prescribing tocolytics contrary to current scientific evidence were not explored in this survey.

More robust research is needed to evaluate the risks and benefits of tocolytics in maintenance tocolysis and repeat tocolysis, as well as in the case of ruptured membranes. Further studies looking at Taiwan-specific economic factors, cultural factors, associated adverse effects, and patient outcomes are warranted in order to understand the complex process of clinician and patient decision making with regards to tocolytic therapy.

## 5. Conclusions

Tocolysis is widely accepted in Taiwan, including in cases with less robust evidence, such as a short cervix, maintenance tocolysis, and repeat tocolysis. More research is needed in terms of patient outcomes and adverse effects to guide clinical practice. Cultural and economic factors also need to be explored to more comprehensively understand the influences that affect the decision making of Taiwanese obstetricians.

## Figures and Tables

**Table 1:** Clinical Scenarios.

Clinical Scenario	Wording in Survey (Translated from Mandarin Chinese)
Abdominal Tightness	The patient came to your office due to continuous abdominal tightness three hours ago. Cardiotocography showed no contractions, and the fetal heartbeat was within normal parameters. Sonography showed a cervical length of 30 mm. There was no vaginal bleeding nor watery discharge. All non-obstetrical causes have been surveyed and excluded. The patient came to your office to ask whether she needs tocolysis.
Short Cervix	The patient had no discomfort, but transvaginal sonography showed cervical length 4 mm. Cardiotocography showed no contractions and the fetal heartbeat was within normal limits. All non-obstetrical causes have been surveyed and excluded. The patient came to your office to ask whether she needs tocolysis.
Preterm Contractions	The patient came to your office due to abdominal pain every 5 min. There was no vaginal bleeding nor watery discharge. Cardiotocography showed regular contractions every 5 min. Transvaginal sonography showed as cervical length of 30 mm. All non-obstetrical causes have been surveyed and excluded. The patient came to your office to ask whether she needs tocolysis.
Preterm Labor	The patient came to your office due to abdominal pain every 5 min. There was mild vaginal bleeding but no watery discharge. Cardiotocography showed regular contractions every 5 min. The pelvic exam showed a cervical dilation of 2 cm. All non-obstetrical causes have been surveyed and excluded. The patient came to your office to ask whether she needs tocolysis.
Maintenance Tocolysis	The patient had undergone 48 h of tocolytic therapy due to preterm labor (regular contractions and cervical dilation of 2 cm) three days ago and has finished a full course of steroid and MgSO_4_. Currently, she has no discomfort; cardiotocography showed no contractions, and the fetal heartbeat was reactive. There was no vaginal bleeding nor watery discharge. The pelvic exam showed a cervical dilation of 1 cm. Due to personal reasons, she requested to be transferred to another doctor and thus came to your office to ask whether she needs further tocolysis.
Repeat Tocolysis	The patient had undergone 48 h of tocolytic therapy due to preterm labor (regular contractions and cervical dilation of 2 cm) one week ago and has finished a full course of steroid and MgSO_4_ and was successfully discharged. However, she started to feel regular contractions every 5 min about 3 h ago with no vaginal bleeding nor watery discharge. Cardiotocography showed contractions every 10 min; the fetal heartbeat was normal, and the pelvic exam showed a cervical dilation of 2 cm. All non-obstetrical causes have been surveyed and excluded. The patient came to your office to ask whether she needs tocolysis again.
PPROM *	The patient noticed copious watery discharge about one hour ago. Cardiotocography showed no contractions, and the fetal heartbeat was normal. There was no cervical dilation, but obvious pooling of amniotic fluid was noted. All non-obstetrical causes have been surveyed and excluded. The patient came to your office to ask whether she needs tocolysis.
PPROM with contractions	The patient noticed copious watery discharge about one hour ago. Cardiotocography showed contractions every 5 min; the fetal heartbeat was normal. There was no cervical dilation, but obvious pooling of amniotic fluid was noted. All non-obstetrical causes have been surveyed and excluded. The patient came to your office to ask whether she needs tocolysis.
PPROM with preterm labor	The patient noticed copious watery discharge about one hour ago. Cardiotocography showed contractions every 5 min; the fetal heartbeat was normal. There was cervical dilation of 2 cm with obvious pooling of amniotic fluid. All non-obstetrical causes have been surveyed and excluded. The patient came to your office to ask whether she needs tocolysis.

* PPROM, preterm premature rupture of membranes.

**Table 2:** Respondent Demographics.

	*n* (%)
Types of practice (*n* = 77)	
Medical Center	44 (57.1)
Regional Hospital	15 (19.5)
Municipal Hospital	8 (10.4)
Local Clinic	10 (13.0)
Practice years (*n* = 77)	
0–5 years	22 (28.6)
6–10 years	7 (9.1)
11–15 years	10 (13.0)
16–20 years	9 (11.7)
21–25 years	13 (16.9)
>26 years	16 (20.8)

**Table 3:** Respondents’ use of tocolytics.

	*n* (%)
**Abdominal tightness (*n* = 76)**	
Would recommend tocolysis	17 (22.4)
Would not recommend but would prescribe if the patient desired	32 (42.1)
Would not recommend and would not prescribe tocolytics	27 (35.5)
First-line tocolytic:	
Nifedipine (oral form)	19 (25.0)
Ritodrine (oral form)	26 (34.7)
Indomethacin (anal form/oral form)	1 (1.3)
MgSO4 (intravenous route)	0 (0.0)
Ritodrine (intravenous route)	1 (1.3)
Atosiban (intravenous route)	0 (0.0)
Other	1 (1.3)
Second-line tocolytic:	
Nifedipine (oral form)	22 (28.9)
Ritodrine (oral form)	12 (15.8)
Indomethacin (anal form/oral form)	3 (4.0)
MgSO4 (intravenous route)	2 (2.6)
Ritodrine (intravenous route)	4 (5.3)
Atosiban (intravenous route)	0 (0.0)
Other	2 (2.6)
**Short Cervix (*n* = 76)**	
Would recommend tocolysis	46 (60.5)
Would not recommend but would prescribe if the patient desired	3 (4.0)
Would not recommend and would not prescribe tocolytics	27 (35.5)
First-line tocolytic:	
Nifedipine (oral form)	14 (18.4)
Ritodrine (oral form)	15 (19.7)
Indomethacin (anal form/oral form)	1 (1.3)
MgSO4 (intravenous route)	0 (0.0)
Ritodrine (intravenous route)	5 (8.3)
Atosiban (intravenous route)	2 (2.6)
Other	12 (15.8)
Second-line tocolytic:	
Nifedipine (oral form)	13 (17.1)
Ritodrine (oral form)	8 (10.5)
Indomethacin (anal form/oral form)	3 (4.0)
MgSO4 (intravenous route)	3 (4.0)
Ritodrine (intravenous route)	10 (13.2)
Atosiban (intravenous route)	1 (1.3)
Other	4 (5.3)
**Preterm contractions (*n* = 76)**	
Would recommend tocolysis	66 (88.0)
Would not recommend but would prescribe if the patient desired	9 (12.0)
Would not recommend and would not prescribe tocolytics	0 (0.0)
First-line tocolytic:	
Nifedipine (oral form)	33 (44.0)
Ritodrine (oral form)	26 (34.7)
Indomethacin (anal form/oral form)	3 (4.0)
MgSO4 (intravenous route)	0 (0.0)
Ritodrine (intravenous route)	14 (18.7)
Atosiban (intravenous route)	0 (0.0)
Other	0 (0.0)
Second-line tocolytic:	
Nifedipine (oral form)	23 (30.7)
Ritodrine (oral form)	14 (18.7)
Indomethacin (anal form/oral form)	10 (13.3)
MgSO4 (intravenous route)	5 (6.7)
Ritodrine (intravenous route)	17 (22.7)
Atosiban (intravenous route)	2 (2.7)
Other	0 (0.0)
**Preterm labor (*n* = 75)**	
Would recommend tocolysis	71 (94.7)
Would not recommend but would prescribe if the patient desired	3 (4.0)
Would not recommend and would not prescribe tocolytics	1 (1.3)
First-line tocolytic:	
Nifedipine (oral form)	15 (20.0)
Ritodrine (oral form)	7 (9.3)
Indomethacin (anal form/oral form)	1 (1.3)
MgSO4 (intravenous route)	2 (2.7)
Ritodrine (intravenous route)	38 (50.1)
Atosiban (intravenous route)	6 (8.0)
Other	4 (5.3)
Second-line tocolytic:	
Nifedipine (oral form)	16 (21.3)
Ritodrine (oral form)	9 (12.0)
Indomethacin (anal form/oral form)	9 (12.0)
MgSO4 (intravenous route)	15 (20.0)
Ritodrine (intravenous route)	6 (8.0)
Atosiban (intravenous route)	13 (17.3)
Other	1 (1.3)
**Maintenance Tocolysis (*n* = 76)**	
Would recommend tocolysis	46 (60.1)
Would not recommend but would prescribe if the patient desired	20 (26.3)
Would not recommend and would not prescribe tocolytics	10 (13.2)
First-line tocolytic:	
Nifedipine (oral form)	24 (31.6)
Ritodrine (oral form)	25 (32.9)
Indomethacin (anal form/oral form)	0 (0.0)
MgSO4 (intravenous route)	2 (2.6)
Ritodrine (intravenous route)	11 (14.5)
Atosiban (intravenous route)	3 (4.0)
Other	1 (1.3)
Second-line tocolytic:	
Nifedipine (oral form)	18 (23.7)
Ritodrine (oral form)	12 (15.8)
Indomethacin (anal form/oral form	9 (11.8)
MgSO4 (intravenous route)	5 (6.6)
Ritodrine (intravenous route)	9 (11.8)
Atosiban (intravenous route)	6 (7.9)
Other	2 (2.6)
**Repeating Tocolysis (*n* = 75)**	
Would recommend tocolysis	67 (89.3)
Would not recommend but would prescribe if the patient desired	5 (6.7)
Would not recommend and would not prescribe tocolytics	3 (4.0)
First-line tocolytic:	
Nifedipine (oral form)	19 (25.3)
Ritodrine (oral form)	13 (17.3)
Indomethacin (anal form/oral form)	1 (1.3)
MgSO4 (intravenous route)	1 (1.3)
Ritodrine (intravenous route)	32(42.7)
Atosiban (intravenous route)	5(6.7)
Other	1 (1.3)
Second-line tocolytic:	
Nifedipine (oral form)	20 (26.3)
Ritodrine (oral form)	6 (8.0)
Indomethacin (anal form/oral form)	9 (12.0)
MgSO4 (intravenous route)	11 (14.7)
Ritodrine (intravenous route)	10 (13.3)
Atosiban (intravenous route)	13 (17.3)
Other	3 (4.0)
**PPROM (*n* = 75)**	
Would recommend tocolysis	49 (65.3)
Would not recommend but would prescribe if the patient desired	8 (10.7)
Would not recommend and would not prescribe tocolytics	17 (22.7)
First-line tocolytic:	
Nifedipine (oral form)	13 (17.3)
Ritodrine (oral form)	14 (18.7)
Indomethacin (anal form/oral form)	3 (4.0)
MgSO4 (intravenous route)	6 (8.0)
Ritodrine (intravenous route)	17 (22.7)
Atosiban (intravenous route)	2 (2.7)
Other	2 (2.7)
Second-line tocolytic:	
Nifedipine (oral form)	20 (26.7)
Ritodrine (oral form)	4 (5.3)
Indomethacin (anal form/oral form)	0 (0.0)
MgSO4 (intravenous route)	8 (10.7)
Ritodrine (intravenous route)	14 (18.7)
Atosiban (intravenous route)	6 (8.0)
Other	3 (4.0)
**PPROM with contractions (*n* = 75)**	
Would recommend tocolysis	62 (82.7)
Would not recommend but would prescribe if the patient desired	4 (5.3)
Would not recommend and would not prescribe tocolytics	9 (12.0)
First-line tocolytic:	
Nifedipine (oral form)	9 (12.0)
Ritodrine (oral form)	10 (13.3)
Indomethacin (anal form/oral form)	1 (1.3)
MgSO4 (intravenous route)	11 (14.7)
Ritodrine (intravenous route)	30 (40.0)
Atosiban (intravenous route)	4 (5.3)
Other	1 (1.3)
Second-line tocolytic:	
Nifedipine (oral form)	18 (24.0)
Ritodrine (oral form)	5 (6.7)
Indomethacin (anal form/oral form)	2 (2.7)
MgSO4 (intravenous route)	9 (12.0)
Ritodrine (intravenous route)	12 (13.0)
Atosiban (intravenous route)	13 (17.3)
Other	3 (4.0)
**PPROM with preterm labor (*n* = 71)**	
Would recommend tocolysis	58 (81.7)
Would not recommend but would prescribe if the patient desired	3 (4.23)
Would not recommend and would not prescribe tocolytics	10 (14.1)
First-line tocolytic:	
Nifedipine (oral form)	6 (8.5)
Ritodrine (oral form)	7 (9.9)
Indomethacin (anal form/oral form)	1 (1.4)
MgSO4 (intravenous route)	10 (14.1)
Ritodrine (intravenous route)	31 (43.7)
Atosiban (intravenous route)	4 (5.6)
Other	2 (2.8)
Second-line tocolytic:	
Nifedipine (oral form)	20 (28.2)
Ritodrine (oral form)	4 (5.6)
Indomethacin (anal form/oral form)	2 (2.8)
MgSO4 (intravenous route)	10 (14.1)
Ritodrine (intravenous route)	9 (12.7)
Atosiban (intravenous route)	12 (16.9)
Other	2 (2.8)

PPROM: preterm premature rupture of membranes.

**Table 4:** Differences between respondents who would recommend, would not recommend but would still prescribe if the patient desires, and would not recommend tocolysis (*n* = 76).

KERRYPNX	Recommend	Not Recommend but Would Still Prescribe	Not Recommend and Not Prescribe	*p*-Value
**Abdominal Tightness (*n*)**	17	32	27	
Practice Type (%)				0.153
Medical Center	22.7	43.2	34.1	
Regional Hospital	14.3	42.9	42.9	
Municipal Hospital	0.0	37.5	62.5	
Local Clinic	50.0	40.0	10.0	
Practice years (%)				0.855
0–5	13.6	50.0	36.4	
6–10	14.3	42.9	42.9	
11–15	22.2	55.6	22.2	
16–20	22.2	33.3	44.4	
21–25	38.5	38.5	23.1	
>25	25.0	31.3	43.8	
**Short cervix (*n*)**	46	3	27	
Practice Type (%)				0.344
Medical Center	56.8	4.5	38.6	
Regional Hospital	75.6	0.0	21.4	
Municipal Hospital	37.5	0.0	62.5	
Local Clinic	70.0	10.0	20.0	
Practice years (%)				0.082
0–5	77.3	4.5	18.2	
6–10	57.1	0.0	42.9	
11–15	55.6	22.2	22.2	
16–20	55.6	0.0	44.4	
21–25	38.5	0.0	61.5	
>25	62.5	0.0	37.5	
**Preterm contractions (*n*)**	66	9	0	
Practice Type (%)				0.249
Medical Center	90.7	9.3	0.0	
Regional Hospital	78.6	21.4	0.0	
Municipal Hospital	75.0	25.0	0.0	
Local Clinic	100.0	0.0	0.0	
Practice years (%)				0.463
0–5	77.3	22.7	0.0	
6–10	100.0	0.0	0.0	
11–15	88.9	11.1	0.0	
16–20	100.0	0.0	0.0	
21–25	92.3	7.7	0.0	
>25	86.7	13.3	0.0	
**Preterm labor (*n*)**	77	3	1	
Practice Type (%)				0.181
Medical Center	93.0	7.0	0.0	
Regional Hospital	100.0	0.0	0.0	
Municipal Hospital	100.0	0.0	0.0	
Local Clinic	90.0	0.0	10.0	
Practice years (%)				0.630
0–5	90.9	9.1	0.0	
6–10	100.0	0.0	0.0	
11–15	100.0	0.0	0.0	
16–20	100.0	0.0	0.0	
21–25	84.6	7.7	7.7	
>25	100.0	0.0	0.0	
**Maintenance Tocolysis (*n*)**	46	20	10	
Practice Type (%)				0.223
Medical Center	63.6	25.0	11.4	
Regional Hospital	60.0	33.3	6.7	
Municipal Hospital	62.5	0.0	37.5	
Local Clinic	44.4	44.4	11.1	
Practice years (%)				0.136
0–5	40.9	50.0	9.1	
6–10	85.7	14.3	0.0	
11–15	70.0	10.0	20.0	
16–20	66.7	11.1	22.2	
21–25	66.7	8.3	25.0	
>25	62.5	31.3	6.25	
**Repeat Tocolysis (*n*)**	67	5	3	
Practice Type (%)				0.424
Medical Center	84.1	11.4	4.5	
Regional Hospital	100.0	0.0	0.0	
Municipal Hospital	100.0	0.0	0.0	
Local Clinic	88.9	0.0	11.1	
Practice years (%)				0.444
0–5	81.8	13.6	4.5	
6–10	100.0	0.0	0.0	
11–15	100.0	0.0	0.0	
16–20	88.9	0.0	11.1	
21–25	75.0	16.7	8.3	
>25	100.0	0.0	0.0	
**PPROM (*n*)**	49	8	17	
Practice Type (%)				0.791
Medical Center	68.1	9.1	22.7	
Regional Hospital	69.2	154	15.4	
Municipal Hospital	62.5	0.0	37.5	
Local Clinic	55.6	22.2	22.2	
Practice years (%)				0.066
0–5	68.2	9.1	22.7	
6–10	57.1	28.6	14.3	
11–15	88.9	0.0	11.1	
16–20	77.8	0.0	22.2	
21–25	46.2	0.0	53.8	
>25	60.0	26.7	13.3	
**PPROM + contractions (*n*)**	62	4	9	
Practice Type (%)				0.229
Medical Center	79.5	6.8	13.6	
Regional Hospital	92.9	7.1	0.0	
Municipal Hospital	100.0	0.0	0.0	
Local Clinic	66.7	0.0	33.3	
Practice years (%)				0.005
0–5	81.8	13.6	4.5	
6–10	100.0	0.0	0.0	
11–15	100.0	0.0	0.0	
16–20	77.8	0.0	22.2	
21–25	53.8	0.0	46.2	
>25	93.3	6.7	0.0	
**PPROM + preterm labor (*n*)**	58	3	10	
Practice Type (%)				0.250
Medical Center	79.5	4.5	15.9	
Regional Hospital	91.7	8.3	0.0	
Municipal Hospital	100.0	0.0	0.0	
Local Clinic	62.5	0.0	37.5	
Practice years (%)				0.021
0–5	86.3	9.1	4.5	
6–10	100.0	0.0	0.0	
11–15	88.9	0.0	11.1	
16–20	75.0	0.0	25.0	
21–25	50.0	0.0	50.0	
>25	92.9	7.1	0.0	

**Table 5:** Differences between respondents according to medical center/non-center and practicing <15 years and ≥15 years.

KERRYPNX	Recommend	Not Recommend but Would Still Prescribe	Not Recommend and Not Prescribe	*p*-Value
**Abdominal Tightness (*n*)**	*n* = 17	*n* = 32	*n* = 27	
Practice Type (%)				0.953
Medical Center	22.7	43.2	34.1	
Non-center	21.9	40.6	37.5	
Practice Years (%)				0.268
<15	15.8	50.0	34.2	
≥15	28.9	34.2	36.8	
**Short cervix (*n*)**	46	3	27	
Practice Type (%)				0.735
Medical Center	56.8	4.5	38.6	
Non-center	65.6	3.1	31.3	
Practice Years (%)				0.034
<15	68.4	7.9	23.7	
>15	52.6	0.0	47.4	
**Preterm contractions (*n*)**	66	9	0	
Practice Type (%)				0.315
Medical Center	90.7	9.3	0.0	
Non-center	84.4	15.6	0.0	
Practice Years (%)				0.306
<15	84.2	15.8	0.0	
≥15	91.9	8.1	0.0	
**Preterm labor (*n*)**	*n* = 77	*n* = 3	*n* = 1	
Practice Type (%)				0.396
Medical Center	93.0	7.0	0.0	
Non-center	93.8	3.1	3.1	
Practice Years (%)				0.370
<15	92.1	7.9	0.0	
≥15	94.6	2.7	2.7	
**Maintenance tocolysis (*n*)**	46	20	10	
Practice Type (%)				0.782
Medical Center	63.6	25.0	11.4	
Non-center	56.3	28.1	15.6	
Practice Years (%)				0.327
<15	56.4	33.3	10.3	
≥15	64.9	18.9	16.2	
* PPROM (n)	49	8	17	
Practice Type (%)				0.798
Medical Center	68.2	9.1	22.7	
Non-center	61.3	12.9	25.8	
Practice Years (%)				0.500
<15	71.1	10.5	18.4	
≥15	59.5	10.8	29.7	
**PPROM + contractions (*n*)**	*n* = 62	*n* = 4	*n* = 9	
Practice Type (%)				0.669
Medical Center	79.5	6.8	13.6	
Non-center	87.1	3.2	9.7	
Practice Years (%)				0.030
<15	89.5	7.9	2.6	
≥15	75.7	2.7	21.6	
**PPROM + preterm labor (*n*)**	58	3	10	
Practice Type (%)				0.782
Medical Center	63.6	25.0	11.4	
Non-center	56.3	28.1	15.6	
Practice Years (%)				0.085
<15	89.2	5.4	5.4	
≥15	73.5	2.9	23.5	

* PPROM: preterm premature rupture of membranes.

## Data Availability

The data that support the findings of this study are available from the corresponding author upon reasonable request.
